# Novel −75°C SEM cooling stage: application for martensitic transformation in steel

**DOI:** 10.1093/jmicro/dfaa051

**Published:** 2020-09-09

**Authors:** Kaneaki Tsuzazki, Motomichi Koyama, Ryosuke Sasaki, Keiichiro Nakafuji, Kazushi Oie, Akinobu Shibata, Takashi Gondo, Hiroya Miyazaki, Hiroshi Akamine, Minoru Nishida

**Affiliations:** Research Center for Structural Materials, National Institute for Materials Science, 1-2-1 Sengen, Tsukuba, 305-0047, Japan; Faculty of Engineering, Kyushu University, 744 Motooka, Nishi-ku, Fukuoka, 819-0395, Japan; Elements Strategy Initiative for Structural Materials (ESISM), Kyoto University, Yoshida-honmachi, Sakyo-ku, Kyoto, 606-8501, Japan; Elements Strategy Initiative for Structural Materials (ESISM), Kyoto University, Yoshida-honmachi, Sakyo-ku, Kyoto, 606-8501, Japan; Institute for Materials Research, Tohoku University, 2-1-1 Katahira, Aoba-ku, Sendai, 980-8577, Japan; Faculty of Engineering, Kyushu University, 744 Motooka, Nishi-ku, Fukuoka, 819-0395, Japan; Faculty of Engineering, Kyushu University, 744 Motooka, Nishi-ku, Fukuoka, 819-0395, Japan; Faculty of Engineering, Kyushu University, 744 Motooka, Nishi-ku, Fukuoka, 819-0395, Japan; Research Center for Structural Materials, National Institute for Materials Science, 1-2-1 Sengen, Tsukuba, 305-0047, Japan; Elements Strategy Initiative for Structural Materials (ESISM), Kyoto University, Yoshida-honmachi, Sakyo-ku, Kyoto, 606-8501, Japan; Mel-Build Corporation, 2-11-36 Ishimaru, Nishi-ku, Fukuoka, 819-0025, Japan; Mel-Build Corporation, 2-11-36 Ishimaru, Nishi-ku, Fukuoka, 819-0025, Japan; Faculty of Engineering Sciences, Kyushu University, Kasuga, Fukuoka, 816-8580, Japan; Faculty of Engineering Sciences, Kyushu University, Kasuga, Fukuoka, 816-8580, Japan

**Keywords:** *in situ* electron microscopy, cooling stage, martensitic transformation, steel, SEM, ECCI

## Abstract

Microstructural changes during the martensitic transformation from face-centred cubic (FCC) to body-centred cubic (BCC) in an Fe-31Ni alloy were observed by scanning electron microscopy (SEM) with a newly developed Peltier stage available at temperatures to  −75°C. Electron channelling contrast imaging (ECCI) was utilized for the *in situ* observation during cooling. Electron backscatter diffraction analysis at ambient temperature (20°C) after the transformation was performed for the crystallographic characterization. A uniform dislocation slip in the FCC matrix associated with the transformation was detected at −57°C. Gradual growth of a BCC martensite was recognized upon cooling from −57°C to −63°C.

*In situ* scanning electron microscopy (SEM) analysis has been recently utilized for martensitic transformation observations in Ti-Ni shape memory alloys [1–4]. Soejima *et al.* [1] have reported the thermoelastic martensitic transformation in Ti–50.8 at% Ni alloy from B2 to B19ʹ structures, in which the first martensite plate that appeared upon cooling disappeared last upon heating in the exact reverse order. Martensitic transformation is a fundamental property of steel [5–7], and important for both the shape memory effect [8,9] and the transformation-induced plasticity effect [10, 11]. Hence, *in situ* observations of the transformation behaviour give us valuable information for further development of high-performance steels. However, no *in situ* SEM studies using a cooling stage have been reported for martensitic transformation in steel. One of the reasons may be the limited working temperature range of the conventional cooling stages. In this report, we introduce applicability of SEM-electron channelling contrast imaging (ECCI) analysis, which is available for dislocation-resolved *in situ* characterization [12, 13], for examining the early stage of the transformation during cooling in steel by using a newly developed Peltier stage (Mel-Build Corp.).

The present Peltier stage can provide a sample temperature as low as −75°C, which is significantly lower than the lowest working temperature of conventional Peltier stages (−50°C) [1–4]. The stage covers the temperature range between −75°C and +80°C, with a temperature control accuracy of ±0.1°C. The maximum cooling rate available in the present condition was 20°C/min. The temperature was detected in one location with a resistance temperature detector. As a further merit, the developed stage possesses a considerably light weight of 125 g, thus enabling us to tilt the SEM stage for electron backscatter diffraction (EBSD) analysis without an exchange of the stage. An overview of the Peltier cooling stage with the temperature detection location and its further performance development are available in the supplemental data (S4. Further development of the Peltier cooling stage).

We have selected an Fe-31Ni (wt. pct.) alloy as a model sample that exhibits face-centred cubic (FCC)-body-centred cubic (BCC) martensitic transformation at temperatures below −50°C [14]. The mean grain size including annealing twin boundaries, measured via line interception method, was 22 μm [14]. The martensitic transformation start temperature (*M_s_*) detected by differential scanning calorimetry (DSC) was −54°C, while the reverse transformation finish temperature (*A_f_*) was 430°C. A sample with 3-mm square and 1-mm thickness was cut from a heat-treated sheet and then mechanically and electrochemically polished. Before SEM observation, the sample was first cooled to −55°C below *M_s_* to produce an amount of martensite plates through a burst type transformation [5] and then heated up to 500°C (above *A_f_*). This treatment had been intentionally performed with the DSC equipment to introduce numerous dislocations and residual stresses into the sample through the martensitic forward and reverse transformations. The sample surface was mechanically and mechanochemically polished before the SEM observation. More detailed information about the alloy and sample preparation is available in the supplemental data (S1. Sample preparation). The dislocations containing sample was cooled down to −62.6°C in a SEM (Carl Zeiss，Merlin). ECCI was operated at an acceleration voltage of 30 kV and a probe current of 10 nA at a working distance of 3.5 mm. The sample was cooled manually upon this observation while checking temperature stability. To reduce the temperature gradient of the 1-mm-thick SEM sample, the average cooling step was maintained at −1.5°C, and the sample was kept at a constant temperature for approximately 15 min through each ECCI observation. The frame rate of the ECCI observation was 1.3\,min (78 sec). The dwell time, the frame rate divided by the number of pixels of the image, was approximately 100 ms (the number of pixels: 1024 × 768 = 786 432). The temperature control accuracy of the present cooling system was within an error of ± 0.1°C. EBSD observation at ambient temperature (20°C) after the cooling test was conducted at 20 kV and 10 nA with a beam step size of 50 nm. The Orientation Imaging Microscopy (OIM) ver. 7.x was used for the analysis.

Figure 1 shows SEM images before and after cooling: (a) is an electron channeling contrast (ECC) image taken before cooling and (b) is the EBSD normal direction-inverse pole figure (ND-IPF) map taken in the same region as in (a) at 20°C, after cooling to −62.6°C. Surface martensite is recognized through different contrast and morphology in Fig. 1a and confirmed by EBSD in Fig. 1b and Fig. S3 (in the supplemental data). The surface martensite might have been introduced during mechanical polishing in the final sample preparation procedure. Fig. 1b indicates that two variants of BCC martensite formed in the grain interior upon cooling. Crystallographic orientations of the martensite plates and the austenite matrix are presented in Fig. 1c. Further EBSD analysis in conjunction with Fig. S3 has confirmed that the pink-coloured region in Fig. 1b corresponds to the annealing twin of the austenite matrix, along with the twin boundaries being a Σ3 coincidence boundary and parallel to 111 traces. The area (d) highlighted in Fig. 1a and 1b was selected for further *in situ* ECCI, due to it partially containing the annealing twin boundary and surface martensite and was therefore expected to exhibit particular properties owing to pre-existing lattice defects and internal stresses. Fig. 1d is the ECC image taken at −20.7°C during the *in situ* observation, where multiple dislocations are seen. It should be noted that these dislocations had been already observed at 20°C before cooling, with nothing happening during cooling down to −20.7°C. Note that the exact *g* vector and the deviation parameter cannot be confirmed in the present observation because the sample was set on different holders for the ECCI and EBSD. However, according to the pole figures shown in Fig. 1c and Fig. S5 (supplemental data), the *g* vector was estimated to be [−11-1]. During further cooling to −62.6°C, motion of an isolated dislocation was detected (Fig. 2), and formation of many slip lines were observed due to the martensitic transformation along with gradual growth of a BCC martensite (Fig. 3). Two frame-by-frame movies of the *in situ* observations are available in the supplemental data (Figs. S1 and S2). Note that we have adjusted the identical observation area of each figure by fitting the location of the annealing twin boundary as an immobile marker, so that dislocation motion could be discussed.

**Fig. 1.**
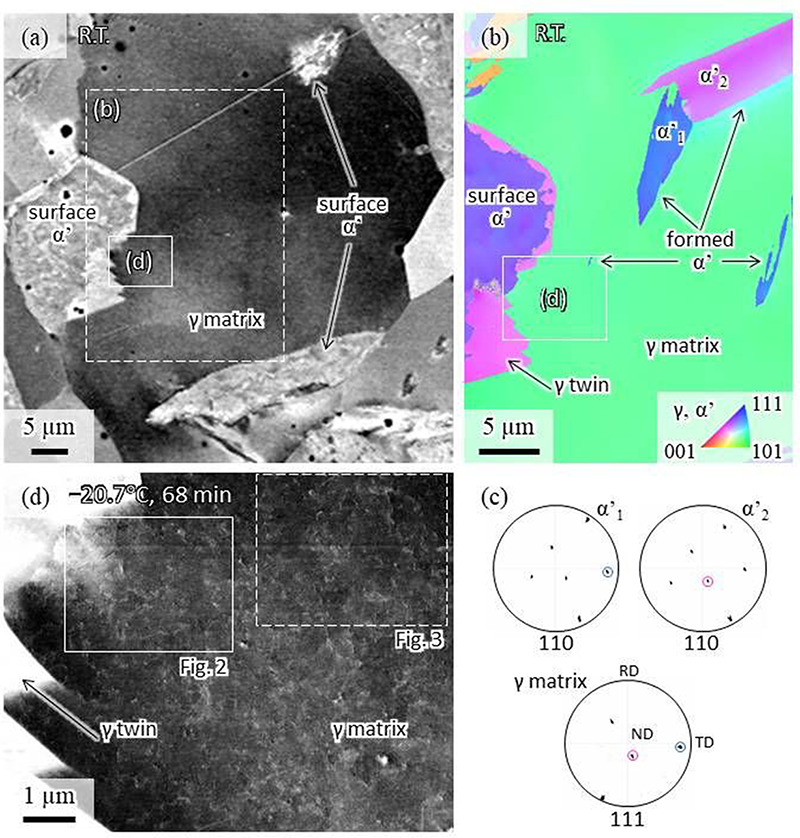
SEM images including the *in situ* observation area before and after cooling: (a) an ECC image taken before cooling, (b) the EBSD ND-IPF map taken in the same region shown in (a) at 20°C after cooling to −62.6°C. (c) Pole figures for the martensite plates (α’_1_ and α’_2_) and the austenite matrix and (d) the ECC image taken at −20.7°C under the *in situ* observation. Note that 68 min is the total experimental time from the cooling initiation when the ECCI image was taken. The white arrow in (b) indicates the small blue martensite that shows gradual growth in Fig. 3. More information about IPF maps and pole figures are available in the supplemental data (Figs. S3 and S4).

Figure 2 shows the result of *in situ* observation during cooling from (a) −32.0°C to (c) −35.7°C. The observation area is indicated in Fig. 1d. The time value on each figure indicates the total experimental time from the cooling initiation when the ECCI image was taken. Two arrows on each figure indicate the identical positions for clarity. An isolated and curved dislocation with a length of approximately 400 nm indicated by the arrows can be recognized with white contrast. The dislocation is seen to move about 50 nm to the lower right during cooling from −32.0°C to −33.8°C (Fig. 2a, b), with subsequent movement upon cooling to −35.7°C (Fig. 2c). It is noteworthy that the dislocation contrast changed to a set of black and white, while its shape from a relatively curved one was altered to a straight line. The straight line in Fig. 2c is parallel to the 111 _FCC_ traces and contains a short dislocation with white contrast at each end. These observations indicate that the straight line may be a slip line on the specimen surface formed by emission of a portion of the dislocation loop to the surface. However, we are not able to discuss the origin of the channelling contrast of the straight line at present and need further information such as the dislocation Burgers vector and the conditions of a surface contamination film. More examples of dislocation motion and slip line formation are available in the movie of Fig. S1 (supplemental data). It should be noted that martensitic transformation was not observed in this region during further cooling to −62.6°C, with the reason for the dislocation motion seen in Fig. 2 not being clear at present. Our estimations converge on the stress caused by the formation of martensite at the subsurface, as a contributing factor; however, we could not confirm in the present test. We can conclude from the result in Fig. 2 that a dislocation-resolved *in situ* observation is applicable with the present Peltier cooling stage system, possessing stability against the mechanical/thermal stage drift.

**Fig. 2.**
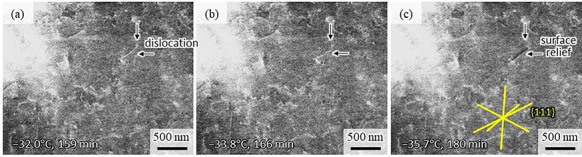
ECC images taken under the *in situ* observation showing a dislocation motion followed by formation of a slip line on the sample surface. The images were taken at (a) −32.0°C, (b) −33.8°C and (c) −35.7°C. The time on each image indicates the total experimental time from the cooling initiation when the image was taken. Two arrows on each figure indicate the identical positions for clarity.

Figure 3 shows ECC images taken during further cooling from (a) −56.1°C to (d) −62.6°C. The observation area is indicated in Fig. 1d. No significant change was observed in this area during cooling to −56.1°C (Fig. 3a) from −20.7°C (Fig. 1d). The change occurred at −56.9°C (Fig. 3b). Numerous long and straight lines are observed, along with the formation of a BCC martensite at the top end of the observation area. The straight lines present considerably uniform spacing, with three different orientations parallel to 111_FCC_ traces, which are indicated by yellow lines in Fig. 3b. This feature indicates that those straight lines were slip traces introduced due to plastic accommodation accompanying martensitic transformation [5, 15, 16]. The contrast of the straight lines during further cooling (Fig. 3c: −57.8°C, 338 min/Fig. 3d: −62.6°C, 431 min) diminished due to contaminations such as hydrocarbons. Another notable observation during further cooling was a gradual growth of the BCC plate. Note that the positions of the plate tip at −56.9°C and −57.8°C are indicated in Fig. 3d, showing a lengthening of approximately 500 nm during cooling. Thickening of the BCC plate is also recognized by comparing Fig. 3d with Fig. 3b. This BCC martensite corresponds to the small blue martensite indicated by the white arrow in Fig. 1b and Fig. S3c (supplemental data). The gradual growth process of the martensite can be more clearly seen in the movie of Fig. S2. Here it must be noted that a burst type transformation to lenticular martensite is well known to occur in Fe-31Ni alloy [14]. The martensite plate shown in Fig. 3 and S2 is small in size, and its morphology unlike lenticular martensite; hence, it is probable that the gradual growth observed in this study is associated with surface martensite forming at a temperature higher than ‘interior’ martensite [5]. However, the mechanisms of gradual growth are not clear at present.

**Fig. 3.**
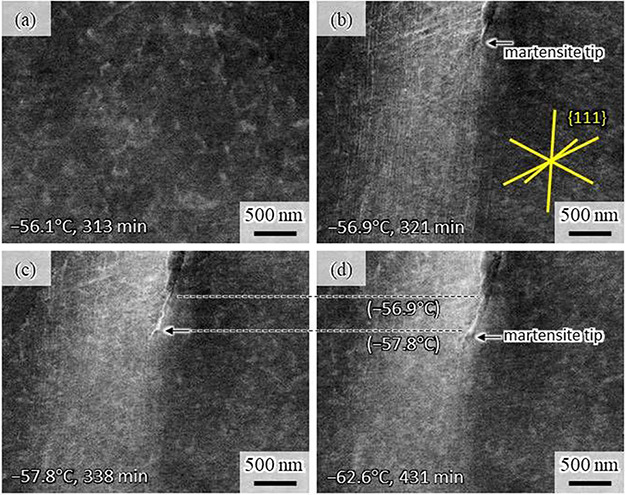
ECC images taken at (a) −56.1°C, (b) −56.9°C, (c) −57.8°C and (d) −62.6°C during the *in situ* observation. The time on each image indicates the total experimental time from the cooling initiation when the image was taken. Formation of many slip lines in the FCC matrix is detected in (b) and gradual growth of a BCC martensite is seen in (b) to (d). The phase map corresponding to (d) is available in the supplemental data (Fig. S3).

In summary, the present study has confirmed that *in situ* ECCI using the newly developed Peltier stage is applicable for characterization of martensitic transformation in steel, even though a further study is needed to evaluate the dislocation motion and gradual growth of martensite observed in the Fe-31Ni alloy. This confirmation encourages us to utilize the present Peltier stage for *in situ* observation of isothermal transformation of lath martensite typically observed at sub-zero temperatures (around −40°C) in Fe-20%Ni-5%Mn alloy [15, 17], because of the engineering significance of lath martensite in heat-treated high-strength steels [6,7,18,19].

## Supplementary Material

dfaa051_Supp
