# Supplementary material for: Novel −75°C SEM cooling stage: application for martensitic transformation in steel
Source: Microscopy (Oxf). 2020 Sep 9;70(2):250–4. doi: 10.1093/jmicro/dfaa051 (PMC7989056; doi:10.1093/jmicro/dfaa051)
Supplement: dfaa051_Supp [file dfaa051_supp.zip › Text of the supplementary data_tsuzaki200901_clean.docx]

Supplementary Data

S1. Sample preparation

An Fe-31Ni (wt pct) alloy was used in the present study. The chemical composition of the alloy (wt pct) is C: < 0.002, Si: < 0.001, Mn: < 0.001, P: < 0.001, S:0.0014, Ni: 31.2, and Fe: bal. As-received material was homogenized at 1200 °C for 24 hours in a vacuum and cold rolled with a thickness reduction of 90 pct. Then, the cold-rolled sheet with 1-mm thickness was austenitized at 850 °C for 30 minutes in a vacuum followed by furnace cooling. This heat treatment produced the average grain size of 22 μm. A line interception method was used to obtain the grain size and annealing twin boundaries were counted as a boundary. Then the present sample with 3 mm square and 1 mm thickness was cut from the heat-treated sheet. Further heat treatment and sample preparation before the SEM observation are available in the main text.

S2. Movies of *in situ* observation

Movies corresponding to Fig. 2 and Fig. 3 in the main text are available in Fig. S1 and S2. Time value along with temperature on each image indicates the total experimental time from the cooling initiation when the image was taken. Yellow font of letters is used in Fig. S2 from the temperature just after the start of martensitic transformation (−56.9 °C).

S3. Further EBSD analysis

An ND-IPF map taken at room temperature after the transformation upon cooling was shown in Fig. 1b. Fig. S3 shows the result of further EBSD analysis in the almost same area as Fig. 1b. It is clearly seen in Fig. S3a-c that the BCC martensite shown in Fig. 3 is a part of a small BCC martensite, which has the same variant as the BCC plate labelled by α’_1_ but is much smaller that the plates α’_1_ and α’_2_. Fig. S3d shows a grain reference orientation deviation (GROD) map. The GROD exhibits a transgranular deformation gradient based on the average orientation for each grain [1], and qualitatively corresponds to the plastic strain distribution [2]. The deformation gradient in the FCC matrix near the plates α’_1_ and α’_2_ is large; it is especially large in the FCC matrix in the bottom side of the plate α’_2_. It is noteworthy that the deformation gradient is small in the observation area of Fig. 3. This small deformation gradient is in good agreement with the uniform sip lines recognized in the ECCI observation (Fig.3).

Fig. S4 shows pole figures of the BCC martensite plates α’_1_ and α’_2_ and the FCC austenite matrix shown in Fig. S3c. It is seen that the martensite plates correspond to two different variants of Kurdjumov–Sachs orientation relationship [3,4] and belong to the same Bain group [5], respectively.

S4. Further development of the Peltier cooling stage

An overview image of the present Peltier stage is shown in Fig. S5. The temperature was detected in one location with a resistance temperature detector and the location is indicated by the arrow in Fig. S5. The cooling stage system has been further modified for the target lowest sample temperature of −100 °C by Mel-Build Corporation [6]. Note that −88 °C is currently available so that a continuous cooling and heating test between −88 and +80 °C can be performed with the developed stage system. The temperature control accuracy of the cooling stage system has been also modified and improved from ±0.1 °C to ±0.005 °C so that more precise experiments are practicable now.

As to heating stages for SEM, they have been already utilized for *in situ* observation of the martensitic transformations that occur above room temperature in low-carbon low-alloy steels [7, 8]. The heating stages cover a temperature range from room temperature to 1000 °C or over, and a rapid cooling faster than 100°C/s is available. Hence, *in situ* SEM observation is now applicable for various types of the martensitic transformations in steel.

References

1. Wright S I, M. Nowell M M, Field D P (2011) A review of strain analysis using electron backscatter diffraction. *Microsc. Microanal.* 17: 316–329.
2. Koyama M, Onishi Y, Noguchi H (2017) Characteristics of hydrogen-assisted intergranular fatigue crack growth in interstitial-free steel: role of plastic strain localization. *Int. J. Fract.* 206: 123–130.
3. Kurdjumow G, Sachs G (1930) Über den Mechanismus der Stahlhärtung. *Z. Physik.* 64:325–343.
4. Dahmen U (1982) Orientation relationships in precipitation systems. *Acta Metall.* 30: 63–73.
5. Takayama N, Miyamoto G, Furuhara T (2012) Effects of transformation temperature on variant pairing of bainitic ferrite in low carbon steel. *Acta Mater.* 60: 2387–2396.
6. [https://www.melbuild.com/cooling-stage.php](about:blank)
7. Nambu S, Shibuta N, Ojima M, Inoue J, Koseki T, Bhadeshia H.K.D.H. (2013) In situ observations and crystallographic analysis of martensitic transformation in steel. *Acta Mater.* 61: 4831-4839.
8. Takayama A, Amino T, Hata K, Miki J (2020) Development of in-situ orientation mapping and microstructure observation system for ferrite/austenite and martensitic transformations in steel. *ISIJ Inter.* 60:714-720.

Figure captions

Fig. S1. Movie of the *in situ* ECCI corresponding Fig. 2 in the main text. It shows more clear examples of dislocation moving followed by slip line formation. The time on each image indicates the total experimental time from the cooling initiation when the image was taken.

Fig. S2. Movie of the *in situ* ECCI corresponding Fig. 3 in the main text. It shows formation of many slip lines in the FCC austenite matrix and a part of small BCC martensite at −56.9 °C and gradual growth of the martensite under further cooling to −62.6 °C. The time on each image indicates the total experimental time from the cooling initiation when the image was taken.

Fig. S3. EBSD images taken at room temperature after cooling to −62.6 °C. (a), (b): phase maps including the area of the *in situ* observation shown in Fig.3. (c) IPF map for surface normal orientations in the almost same area as Fig. 1b. (d) GROD map taken in the region indicated by a rectangle in Fig. S4c.

Fig. S4. Pole figures of 100, 111, and 110 poles for the FCC austenite matrix and the BCC martensite plates α’_1_ and α’_2_, indicating that the martensite plates hold near Kurdjumov–Sachs orientation relationship with the austenite matrix and belong to the same Bain group.

Fig. S5. Overview image of the newly developed Peltier cooling stage (unit: mm).
