# Supplementary figures and images for: Novel −75°C SEM cooling stage: application for martensitic transformation in steel
Source: Microscopy (Oxf). 2020 Sep 9;70(2):250–4. doi: 10.1093/jmicro/dfaa051 (PMC7989056; doi:10.1093/jmicro/dfaa051)

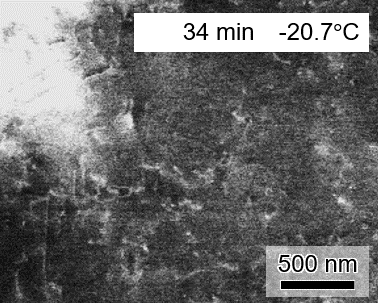

Supplement: dfaa051_Supp [file dfaa051_supp.zip › Fig. S1_Movie of dislocation motion.gif]

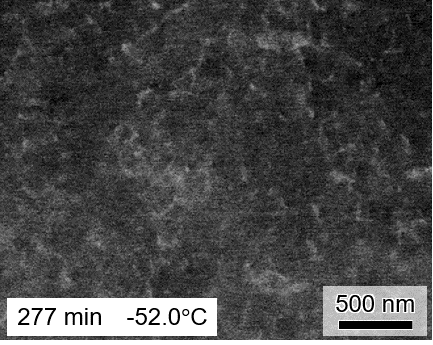

Supplement: dfaa051_Supp [file dfaa051_supp.zip › Fig. S2_Movie of mastenisite growth.gif]
